# DECREASED SURGICAL DURATION, LESS COMPLICATIONS, AND FASTER RETURN TO ACTIVITIES ACROSS THE LEARNING CURVE FOR THE ARTHROSCOPIC LATARJET TECHNIQUE

**DOI:** 10.1590/1413-785220243205e277567

**Published:** 2024-10-28

**Authors:** Wagner Castropil, Juliana Ribeiro Mauad, Fernando Henrique Barcelos Amorim, Alexandre Carneiro Bitar, Antonio Guilherme Padovani Garofo, Breno Schor

**Affiliations:** 1. Instituto Vita, Department of Orthopedic Surgery, Sao Paulo, Brazil.; 2. Hospital Beneficiência Portuguesa, Department of Orthopedic Surgery, Sao Paulo, Brazil.

**Keywords:** Instability, Shoulder, Surgeon Experience, Learning Curve, Instabilidade, Ombro, Experiência do Cirurgião, Curva de Aprendizado

## Abstract

Objetive: This study aims to analyze the learning curves in performing the arthroscopic Latarjet surgery. Methods: This was an observational, retrospective, single-center study. All cases of arthroscopic Latarjet performed in this institution from 2016 to 2021 were included. The data analyzed were surgical time (of the chief surgeon alone and the group of surgeons), complications, and time until the return to sports activities. Technical observations about the learning process were also reported. Results: In total, 50 consecutive cases were included (93% retention of the initial sample identified at the institution). The decrease in surgical time was presented logarithmically and showed a decrease in time both for the individualized analysis of the senior surgeon (r = −0.67, p < 0.001) and for the surgical group (r = −0.476, p < 0.001). Mean operating time (and standard deviation) dropped from 235 minutes (73) in the first 10 cases to 156 minutes (34) for the subsequent cases (p < 0.001). In the first 20 cases, five intercurrences were recorded and three reoperations were performed, whereas subsequent cases presented only one intercurrence requiring surgical intervention (p = 0.032). The median time to return to sport was nine months for the first 20 cases versus six months for subsequent cases (p = 0.001). Conclusion: The learning curve for the arthroscopic Latarjet procedure showed a progressive decrease in operative time, complications, and time to return to sports activities. This suggests that the surgeon developed the necessary skills and confidence to reach a plateau of expertise to perform the surgical procedure. ***Level of evidence IV, Observational retrospective.***

## RESUMO

Objetivo: Analisar a curva de aprendizado para a realização artroscópica da cirurgia de Latarjet. Métodos: Este foi um estudo observacional, retrospectivo e unicêntrico. Foram incluídos todos os casos de Latarjet artroscópico realizados nesta instituição, de 2016 a 2021. Os dados analisados foram: tempo cirúrgico (somente do cirurgião chefe e do grupo de cirurgiões), complicações e tempo até o retorno às atividades esportivas. Observações técnicas sobre o processo de aprendizagem também foram relatadas. Resultados: Foram incluídos 50 casos consecutivos (retenção de 93% da amostra inicial identificada na instituição). A diminuição do tempo cirúrgico foi apresentada de forma logarítmica e mostrou redução do tempo, tanto para a análise individualizada do cirurgião sênior (r = −0,67, p < 0,001) quanto para o grupo cirúrgico (r = −0,476, p < 0,001). O tempo operatório médio (e desvio padrão) caiu de 235 minutos (73) nos primeiros 10 casos para 156 minutos (34) nos casos subsequentes (p < 0,001). Nos primeiros 20 casos foram documentadas cinco intercorrências e realizadas três reoperações, enquanto nos casos subsequentes ocorreu apenas uma intercorrência com necessidade de intervenção cirúrgica (p = 0,032). O tempo médio para retorno ao esporte foi de nove meses para os primeiros 20 casos versus seis meses para os casos subsequentes (p = 0,001). Conclusão: Durante a adoção inicial do Latarjet artroscópico, houve diminuição progressiva do tempo operatório, das complicações e do tempo de retorno às atividades esportivas até que o cirurgião ganhasse a habilidade e a confiança necessárias para atingir o patamar de expertise para realização do procedimento cirúrgico. ***Nível de evidência IV, Retrospectivo observacional.***

## INTRODUCTION

 Anterior glenohumeral dislocation is the most common type of shoulder dislocation, accounting for 90% of cases. It is a potentially disabling injury and frequently affects young athletes, which encourages continuous improvement in the development of techniques to treat this pathology. ^1^

 Considering the high rates of recurrences described, Balg and Boileau designed the Instability Severity Index Score (ISIS), which considers patient’s characteristics, type of activity, and radiological images. Scores below 4 show recurrence rate of approximately 10%, whereas scores greater than or equal to 4 have an approximate recurrence rate of 70%, thus requiring further techniques for repair, with the need for bone blocks, such as the Bristow-Latarjet surgery. ^2^

 The Bristow-Latarjet technique involves osteotomy of the coracoid apophysis and its transfer, along with the conjoined tendon, to address the bone defect in the anteroinferior portion of the glenoid. ^3^ ^,^ ^4^ In 2007, Lafosse et al. first described arthroscopic Latarjet surgery, finding fewer complications compared to open surgery. ^5^

 The literature have suggested that the arthroscopic technique shows potential benefits, including less damage to the adjacent tissues, less postoperative stiffness, and faster rehabilitation. ^6^ However, it is technically challenging due to concerns about potential surgical risks during the initial phase of the learning curve. ^7–9^ Thus, this study aimed to analyze the learning curve for performing arthroscopic Latarjet surgery. 

## MATERIALS AND METHODS

 After approval by the Research Ethics Committee, an observational, retrospective, single-center study of a series of cases was conducted. Screening of the participants was accomplished by searching the medical records of the institution. Inclusion criteria were: individuals who underwent arthroscopic Latarjet surgery from April 2016 to July 2021 to treat anterior glenoid bone defects greater than 20%, an engaging Hill-Sachs lesion with ISIS score greater than four ^10^ , or a failed Bankart repair. Exclusion criteria included the presence of rotator cuff injuries, fractures of the proximal third of the humerus, and/or insufficient medical records for the required analyses. 

The data collected and analyzed were sex, age, surgical time, need for reverting to open surgery, surgical or perioperative complications, reoperation, and time to return to physical activities.

The study was approved by Instituto Fleury under the number 48639121.0.0000.5474.

### Surgical technique

 The arthroscopy was performed with 0.9% saline solution with half an ampoule of adrenaline with vasoconstrictor for each liter of saline. The pressure in the infusion pump in the arthroscope was maintained at 45 to 50 mmHg, and 1 g of tranexamic acid was administered during anesthetic induction. Arm support was provided by an articulated mechanical arm (Trimano) and bipolar radiofrequency was used. The arthroscopic portals were used as recommended by Lafosse ^5^ and as previously described. ^11^

 Once the kit for performing the arthroscopic Latarjet technique became available in our country, the institution where this research was conducted began to treat cases using this surgical technique. A senior surgeon from the group took the lead as head surgeon in the first cases. To ensure the safety of the coracoid osteotomy and its positioning, the first 10 cases were completely performed using the arthroscopic technique with subsequent open surgery for checking and making final adjustments when necessary, as previously suggested for this surgical technique ^12^ . The patients were previously informed that the arthroscopic and open surgery were going to be programmed and performed. 

As the surgical group gained experience, after the first 10 cases, the procedure was completely performed arthroscopically and other surgeons began to take on their first cases as the lead surgeon, accompanied by the senior surgeon.

### Data analysis

A descriptive and inferential presentation of the data was prepared. Technical observations about the learning process were compiled in descriptive form.

For learning curve, three models were considered: (A) chronological order with only the senior surgeon cases, (B) chronological order with all group cases, and (C) order considering the number of cases for each surgeon individually. The learning rate was calculated for model (A) using the learning curve data for the single surgeon cases only. The Kolmogorov-Smirnov test was used to evaluate the normality of the data. Parametric correlation analysis (Pearson’s correlation) between the case number and the surgical time was conducted using the least squares method (logarithmic transformation of the variables involved). Group case series were divided into five consecutive strata in chronological order and the mean surgical times of the blocks were compared using analysis of variance (ANOVA) and the two-sample Student’s t-test to evaluate change between the blocks during the learning process. A 2×2 contingency table was used with Pearson’s test for the comparison between the blocks of cases with or without complications. Moreover, the Mann-Whitney test was used to compare the times to return to physical activities. The SPSS Statistics 22 (IBM®) software program was used for data analysis, and the significance level was set at 0.05.

## RESULTS

From the initial sample of 54 identified cases, we obtained the necessary data for 50 individuals (93% retention). In total, 44 patients were male (88% of the total), and the mean patient age was 32 years (standard deviation, SD 11). A total of 34 surgeries were performed by the primary author as the lead surgeon. The other procedures were performed by four other surgeons in the group as the leads, but always with the primary author as the supervisor.

**Figure 1.**
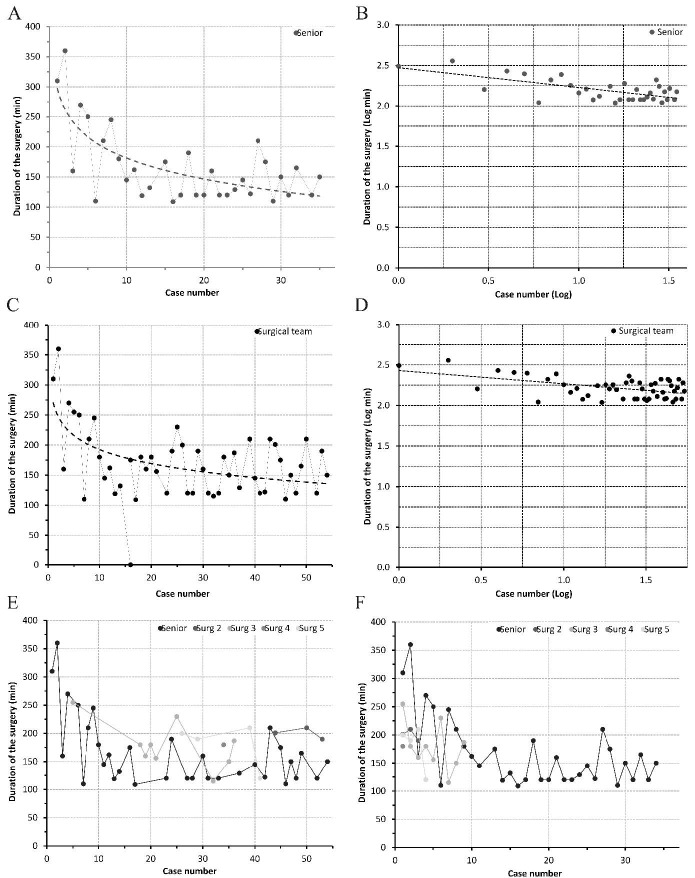
Learning curves created by plotting surgical time by case number. (a) Learning curve of the senior surgeon with raw data and in (b) logarithmic transformation. The learning curve was estimated as 84% (2−0.163). (c) Learning curve of all cases of the surgical group directed by the senior surgeon with raw data and in (d) logarithmic transformation. The learning curve was estimated as 89% (2−0.244). Graphs A and C show logarithmic trendlines and graphs B and D show linear trendlines. (e) Learning curve of the surgical group in the chronological and cumulative order in which the cases were operated. (f) Learning curve of the different lead surgeons of the same surgical group (the cases were not cumulatively counted among the different surgeons).

Figure 1 shows the operating time spent per number of consecutive cases and the estimated time based on the learning curve model. The decrease in surgical time is evident over the course of consecutive cases across the learning curve for this surgical technique. 

 The correlation model observed between the surgical time (in log) and the secondary evolution of the cases operated only by the senior surgeon demonstrated a significant statistical correlation, in which a decrease in surgical time was observed (Pearson’s linear correlation coefficient, r = −0.67, p < 0.001, Figures 1 a and 1 b). 

 In our analysis of the group learning curve, we also observed a statistically significant decrease in the surgical time over the course of the cases operated (r = −0.476, p < 0.001, Figures 1 c and 1 d). 

**Figure 2.**
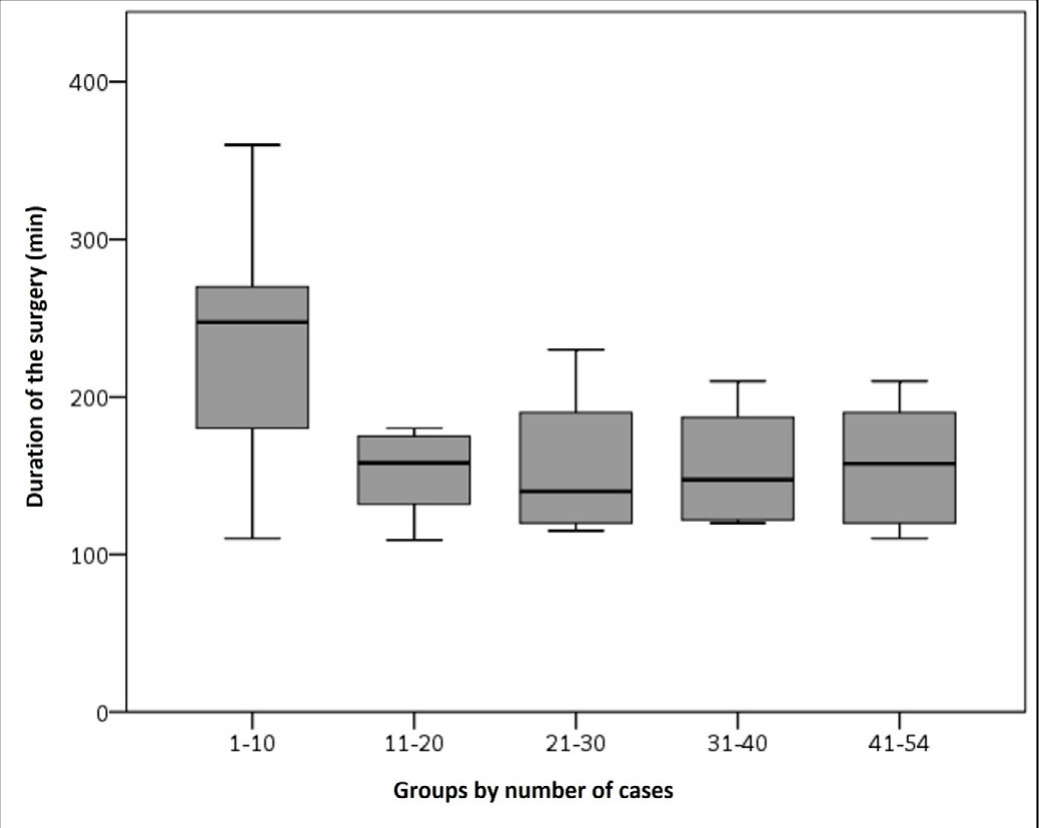
Comparison of surgical times by consecutive groups of cases included in this study. ANOVA (p < 0.001), * Group 1–10 statistically significant in Student’s t-test compared to the other groups (p = 0.006: 1–10 vs. 11–20, p = 0.011: 1–10 vs. 21–30, p = 0.010: 1–10 vs. 31–40, p = 0.011: 1–10 vs. 41–54)

 By separating the cases into consecutive chronological blocks of 10, it was possible to observe that the mean surgical time of the cases after the tenth dropped significantly ( Figure 2 , p < 0.001) from 235 minutes (cases 1–10) to 151 (11–20), 165 (21–30), 150 (31–40), and 157 (41–54). After the tenth case, the variability (standard deviation) of the surgical times dropped from 73 minutes (95% CI 182–287, cases 1–10) to 34 minutes (95% CI 145–167, cases 11–54), showing trends to follow the estimated time curve more faithfully, in a plateau projection. 

In the first 10 cases, a total of five complications were found, with two cases of neurapraxia of the musculocutaneous nerves, with spontaneous resolution, a case of coracoid graft fracture, resolved intraoperatively with graft fixation using a screw, and two cases of grafts in a highly lateralized position, requiring thinning of the graft and removal of the screws. All cases progressed well, without sequelae. In this study, in our analysis of cases 30 to 50, we encountered only one complication. Breakage of the coracoid graft occurred in case 43, which was fixed with only one screw and was resolved intraoperatively, requiring only conversion to open surgery, without complications or future sequelae for the patient. We observed a change in pattern after the 20th case (p = 0.032). The median time to return to physical activities in the first 20 cases was nine months (min.-max., 4–18) versus six months (min.-max., 4–9) for subsequent cases (p = 0.001).

As qualitative observations, we noted some recurring challenges from the first surgical procedure. First, we noticed a high split in the subscapularis muscle, leaving only a narrow upper layer, which could lead to muscle weakness. Another issue was related to the size of the coracoid. During the osteotomy, the graft often was found to be quite small at the base, with excess coracoid, which could cause an internal impact in the future or, if the grafts were smaller than 1 cm, they might not be sufficient to stabilize the humeral head against the glenoid. Moreover, when the graft was fixed arthroscopically, we noted that it was usually positioned excessively at the base of the glenoid and excessively lateralized, which could generate impact and chondral injury on the humeral head.

After the first 10 surgeries, we were able to make the necessary adjustments so that all the procedures were performed solely by arthroscopy without checking via open visualization. This was made possible with the subscapularis split in the proper location, the coracoid tip with the correct size, and the more medialized positioning of the graft, preventing impact with the humeral head and adequately stabilizing the glenohumeral joint.

## DISCUSSION

 In our study, we observed a reduction in the required surgical time, a decrease in the number of complications, and an acceleration of the return to physical activities accompanying the learning curve. These findings corroborate the fact that the arthroscopic Latarjet technique can show favorable clinical results, even better when performed by experienced surgeons. ^6^ ^,^ ^9^

 As previously discussed in the literature, ^12^ ^,^ ^13^ the technical challenges recurrent during a surgeon’s first cases operating via arthroscopic Latarjet surgery should be noted and include the subscapularis split and the preparation and proper positioning of the coracoid graft. ^11^ These challenges can be attributed to the inadequate placement of the portals. For example, while the midsub portal exposes the entire extension of the subscapularis muscle, facilitating a proper split, and the pectoral portal, also known as the “suicidal portal,” provides a good view for positioning the coracoid on the glenoid, these are details that require training and careful attention of the surgeon performing these operations. ^9^ ^,^ ^11^ ^,^ ^14^

 According to Kany et al., 50% of open Latarjet procedures evolve with poor positioning of the bone graft. They also state that, after conducting surgical planning using computed tomography of the shoulder, 81% of the patients who underwent arthroscopic Latarjet had good positioning of the coracoid, which corroborates the fact that graft positioning may be related to surgical planning and adequate reproduction of the arthroscopic Latarjet technique. ^15^ In our study, we had only two cases of poor graft positioning out of 50, which is lower than the values normally found in the literature both for open and arthroscopic Latarjet. The decline in the number of complications from the open technique had already been documented, ^16^ ^,^ ^17^ but unlike other previous reports using the arthroscopic technique, ^7^ ^,^ ^10^ ^,^ ^12^ ^,^ ^18^ the present study was able to also attest to such a decline in the minimally invasive technique. 

 In fact, the negative correlation between surgical time and surgeon’s experience is the most commonly studied outcome in the initial adoption of both the open and arthroscopic Latarjet techniques. ^17^

 In 2018, a systematic review ^17^ estimated a number that can be defined as the case volume necessary to achieve proficiency in the arthroscopic procedure. By grouping the data from three studies ^10^ ^,^ ^12^ ^,^ ^18^ , they observed that the surgical time was greater in the 1st to 42nd cases than in the subsequent cases (43–105). In a study with 12 surgeons in five different countries ^9^ , the authors concluded that, with a high volume of cases, surgeons reach a learning curve plateau at around 30 to 50 cases. 

 After that review article, and in addition to a mere decline, in 2020, Getz and Joyce concluded that the arthroscopic Latarjet procedure, after a learning curve of 20 to 25 patients, may be advantageous over the open procedure by reducing the time to return to sports, scar size, and joint stiffness, in addition to showing no statistical difference in relation to the number of complications. ^19^ Surgical time and incidence of complications were found to significantly decreased after the first 25 and 30 cases in two other studies. ^20^ ^,^ ^21^

 In this article, we observed a decrease in the three outcomes studied starting with the 21st case, a result consistent with the findings in a more recently study published by Leuzingher et al. ^7^ We highlight that the surgeon was already familiar with the technique, as has completed observational internships and undergone training, which may have optimized the adoption of the technique. 

 When comparing the open and arthroscopic techniques, there are no significant differences in terms of complications or outcomes, although it is necessary to traverse the arthroscopic surgery learning and experience curve. ^22^ A limitation related to the arthroscopic Latarjet technique is the increased total cost of the surgery since it requires specific instruments, which can limit its use. ^22^ Arthroscopic Latarjet surgery is a highly complex technique; however, when performed by surgeons with extensive experience in arthroscopy and shoulder surgery, it is reproducible and safe, as well as advantageous, especially for athletes and sportspersons who want an earlier return to sports. ^19^ ^,^ ^22^ As far as we know, this was the first article documenting the reduced time to return to sports activities. 

 This work was a retrospective study that analyzed the initial learning curve for the arthroscopic Latarjet technique performed by a senior surgeon and a surgical team. A long-term analysis of these learning curves may be important to determine whether the observed plateau trend in surgical time will be sustained. In this article, clinical outcomes across the learning curve were not studied, but a previous study found no significant differences in Walch-Duplay scores, Rowe scores, or patient satisfaction levels. ^9^

## CONCLUSION

In conclusion, during the initial adoption of the arthroscopic Latarjet procedure, we observed a progressive decrease in surgical time, a reduction in the number of complications, and a shorter time before patients were allowed to return to sports activities. As surgeons progress in this learning curve, they become more familiar with the procedure, overcome technical difficulties, and develop the skills and confidence necessary to optimally perform the surgical treatment.
